# Linking Chromosomal Silencing With *Xist* Expression From Autosomal Integrated Transgenes

**DOI:** 10.3389/fcell.2021.693154

**Published:** 2021-06-18

**Authors:** Ikrame Naciri, Benjamin Lin, Chiu-Ho Webb, Shan Jiang, Sarah Carmona, Wenzhu Liu, Ali Mortazavi, Sha Sun

**Affiliations:** Department of Developmental and Cell Biology, School of Biological Sciences, University of California, Irvine, Irvine, CA, United States

**Keywords:** *Xist*, X-chromosome inactivation, transgene integration, gene silencing, chromosome conformation

## Abstract

*Xist* is the master regulator of X-Chromosome Inactivation (XCI), the mammalian dosage compensation mechanism that silences one of the two X chromosomes in a female cell. XCI is established during early embryonic development. *Xist* transgene (Tg) integrated into an autosome can induce transcriptional silencing of flanking genes; however, the effect and mechanism of *Xist* RNA on autosomal sequence silencing remain elusive. In this study, we investigate an autosomal integration of *Xist* Tg that is compatible with mouse viability but causes male sterility in homozygous transgenic mice. We observed ectopic *Xist* expression in the transgenic male cells along with a transcriptional reduction of genes clustered in four segments on the mouse chromosome 1 (Chr 1). RNA/DNA Fluorescent *in situ* Hybridization (FISH) and chromosome painting confirmed that *Xist* Tg is associated with chromosome 1. To determine the spreading mechanism of autosomal silencing induced by *Xist* Tg on Chr 1, we analyzed the positions of the transcriptionally repressed chromosomal sequences relative to the *Xist* Tg location inside the cell nucleus. Our results show that the transcriptionally repressed chromosomal segments are closely proximal to *Xist* Tg in the three-dimensional nucleus space. Our findings therefore support a model that *Xist* directs and maintains long-range transcriptional silencing facilitated by the three-dimensional chromosome organization.

## Introduction

In 1961, Marie France Lyon proposed that in mammals, one of the two X chromosomes in females is inactivated to offset the difference in X-linked gene dose between male and female (Lyon, 1961). Such a dosage-compensation strategy is called X-chromosome inactivation (XCI). Cumulative studies have been conducted seeking to unravel the molecular mechanisms involved in XCI. One of the key factors of this process is the long non-coding RNA *Xist*—*X*-*i*nactive *s*pecific *t*ranscript, which is the necessary and master regulator of XCI in both mouse and human (Brockdorff et al., 1992; Brown et al., 1992). *Xist* is located on the X chromosome in the *X inactivation center* (*Xic*) locus that contains a cluster of five non-coding RNA genes, *Ftx*, *Jpx*, *Xist*, *Tsix*, and *Tsx*, which are involved in regulating XCI in eutherian mammals (Brockdorff et al., 1992; Brown et al., 1992; Wutz et al., 2002; Payer and Lee, 2008; Sun et al., 2013; Furlan et al., 2018). XCI occurs during early embryogenesis with the transcription of *Xist* and the upregulation of *Xist* RNA, which initiates a subsequent epigenetic modification and a *cis*-spreading of gene silencing across the future inactive X chromosome (Brockdorff et al., 1992; Brown et al., 1992; Marahrens et al., 1997). *Xist* RNA is also capable of inducing chromosomal silencing when it is ectopically transcribed from a transgene in an autosome, recapitulating the XCI effects outside the X chromosome (Lee and Jaenisch, 1997; Okamoto et al., 2005; Sun et al., 2015).

The efficacy of shutting down an entire chromosome addresses not only the genomic dosage imbalance of the sex chromosome, but also the chromosomal aneuploidies that have been observed as common causes for cellular defects during early embryogenesis. Indeed, *Xist*-mediated inactivation mechanisms have been applied for a potential “chromosome therapy” of Down syndrome (Jiang et al., 2013). Down syndrome is a genetic disorder derived from trisomy of chromosome 21, which induces intellectual deficiency, congenital heart defects, Alzheimer’s disease and other related health problems (Dierssen, 2012). By using zinc finger nucleases genome editing, a large inducible *Xist* Tg was integrated into chromosome 21 to silence genes on the extra chromosome in induced pluripotent stem (iPS) cells for human trisomy 21, which showed that transgenic *Xist* RNA can function on a human autosome for large-scale chromosome silencing. Importantly, the impairment proliferation associated with the overexpression of chromosome 21 and the neural rosette formation that are ameliorated upon induction of the *Xist* Tg point to a promising new treatment of Down syndrome (Jiang et al., 2013). However, such *Xist*-mediated silencing in autosomes can be highly variable in its extent of silencing and may be dependent on the transgene integration site, suggesting the involvement of other interacting molecules and chromosomal contacts in chromosome silencing (Kelsey et al., 2015). In short, the extent of *Xist*-mediated gene silencing on autosomes and the factors determining the silencing effects remain to be elucidated.

Here, we examine a specific case of *Xist* Tg integration in the mouse autosome. We used fluorescent-labeled DNA probes for localization of the *Xist* Tg in the cell nucleus, combined with RNA-sequencing for transcriptome profiling of the transgenic mouse cells, which allowed us to pinpoint the autosomal gene silencing and the integration site of the *Xist* Tg. By analyzing the nuclear distance between the *Xist* Tg and the autosomal genes, we determined the silencing effects of the ectopic *Xist* RNA in the three-dimensional space of the cell nucleus. The combined data of RNA-seq and fluorescent *in situ* hybridization thus elucidate the chromosome silencing effects induced by transgenic *Xist* expression from an autosomal site in mouse cells.

## Materials and Methods

### Mouse Fibroblast Cells

TgBAC8 mouse lines were generated and described previously as transgenic mice carrying 200 kb of sequence from *Xic* including *Xist* (Sun et al., 2015). C57BL/6J-TgBAC8-2087 line was used in this study, for which Tg2087/+ mice were crossed to wildtype C57B1/6J mice to obtain offspring that contain littermates of transgenic (Tg2087/+) and wildtype (+/+) animals. Tail tip fibroblasts (TTF) were isolated from tail tip tissues of transgenic and wildtype mice from the same litter and grown in DMEM medium containing 10% FBS supplemented with MEM non-essential amino acids, 25 mM HEPES pH 7.2–7.5, 0.1 mM 2-mercaptoethanol, penicillin, and streptomycin. TTFs were then immortalized with Simian virus 40 large T cDNA following the protocol as previously described (Brown et al., 1986). Transgenic samples and wildtype (control) samples were processed in parallel following the same procedure.

### RNA Preparation

Total RNA was extracted from cells grown on a 15 cm tissue culture plate using Trizol (Thermo Fisher Scientific), followed by DNase treatment using TURBO DNA-free^TM^ Kit (Thermo Fisher Scientific). Polyadenylated mRNA was isolated from total RNA using Dynabeads^TM^ mRNA Purification Kit (Thermo Fisher Scientific). RNA integrity was checked by Bioanalyzer (Agilent 2100), followed by fragmentation with Mg2+ -catalyzed hydrolysis.

### cDNA Library Construction

cDNA library was constructed as previously described (Mortazavi et al., 2008). In brief, the first-strand cDNA was reverse-transcribed with random primers, followed by deoxyadenosine (dA) addition and adaptor ligation. Then, an RNA gel-purified was performed for size selection of 300 bp, followed by UNG treatment for strand specificity and PCR amplification.

### High Throughput Sequencing

cDNA sequencing was performed as previously reported (Mortazavi et al., 2008). DNA quality and size were checked by Bioanalyzer (Agilent 2100), quantified by KAPA Library quantification Kit (Illumina), followed by sequencing on NextSeq500 (Illumina) using paired-end mode. The sequencing data have been deposited in NCBI’s Gene Expression Omnibus and are accessible through GEO Series Accession number GSE 156393.

### Data Analysis

Analysis of RNA-seq data was performed with the Bioconductor package Rsubread (index building, alignment with genome mm10). The count table was generated with the feature count function in R and the counts were converted into FKPM by using the R package “count to FPKM.” For differential gene expression, we used the package DESeq.

### Fluorescence *in situ* Hybridization (FISH)

DNA and RNA FISH were performed with probes generated using BAC clones as DNA templates (Sun et al., 2013). Mouse chromosome 1 BAC clones were obtained from the CHORI BACPAC Resources Center^1^ with the following ID numbers: RP24-402L7 (Segment A); RP24-159O10 (Segment B); RP24-232L18 (Segment C); RP23-2C9 (Segment E). Fluorescent Cyanine and Fluorescein probes were then generated using a Nick Translation Kit (Roche) supplemented with either Fluorescein-12-dUTP or Cyanine-3-dUTP (Enzo) according to the manufacturer’s protocol. Unincorporated nucleotides were removed using G-50 columns (GE). FISH probes were ethanol precipitated and resuspended in 50% formamide, 2xSSC pH7.4, 10% dextran sulfate, 1 mg/ml BSA, and 100 ng/μl mouse Cot-1 DNA. Transgenic and wildtype TTFs were grown on gelatin treated 10-well microscope slides (Fisher Scientific). Cells were permeabilized in the CSKT buffer at 4°C for 5 min and then fixed in 4% paraformaldehyde at room temperature for 10 min. For RNA FISH, slides were not denatured. For DNA FISH, slides were treated in 400 μg/ml RNaseA at 37°C for 30 min, followed by a PBS wash and a treatment with 0.2 N HCl 0.5% Tween-20 at room temperature for 10 min. The slides were denatured in 70% formamide, 2xSSC pH7.4 at 80°C for 10 min, followed by sequential dehydration in 70, 80, and 100% ethanol at 4°C. FISH probes of 10 ng/μl were denatured at 80°C for 10 min and then kept at 37°C for more than 15 min, and then loaded directly onto cell samples on the slides. With coverslips placed on top of the cells on the slides, hybridizations were carried out in a humidified chamber at 37°C for 16 h. Afterward, slides were washed with 50% formamide, 2xSSC pH7.4 at 37°C for 3 times, and then with 0.1xSSC pH7.4 at 37°C for 3 times. Slides were air dried and mounted in VECTASHIELD Mounting Media (Vecta Labs) with DAPI 1.5 μg/ml. Slides were visualized and imaged with Zeiss LSM780 and LSM700 confocal microscopes, and the images were processed with ZEN Software (Zeiss) and Volocity software (Perkin Elmer).

For dual-color DNA FISH, chromosome paints were purchased as XCyting Mouse Chromosome Painting Probes (MetaSystems): XMP X Orange D-1420-050-OR (Chr X); XMP Y Green D-1421-050-FI (Chr Y); XMP 1 Green D-1401-050-FI (Chr 1); XMP 4 Green D-1404-050-FI (Chr 4); XMP 9 Green D-1409-050-FI (Chr 9). For chromosome and *Xic* localization analysis, Z-section images were captured and analyzed within each individual section across all sections of the Z stack. In particular, co-localization events between the chromosome paint signals and the Xic signals were detected by examining the section images individually in Volocity software. Measurements between chromosomal segments (on chromosome 1) and the transgene were performed with 3D Image Analysis Tools in Volocity software. The BAC probes yield discrete pinpoint spots by DNA FISH and distances are measured by drawing a straight line between the center of each spot. Nuclei with more than two resolvable signals were scored. Cells that displayed two or more signals and the two shortest measurements between the chromosomal segment and the transgene were recorded and the average value was taken for each cell. The normalized distance was obtained by subtracting the median distance measurements across all WT cells from each averaged measurement for each TG cell. Statistical analysis was performed in R Studio.

## Results

### *Xic* Transgene Induces *Xist* Expression and Gene Silencing in an Autosome

**FIGURE 1:**
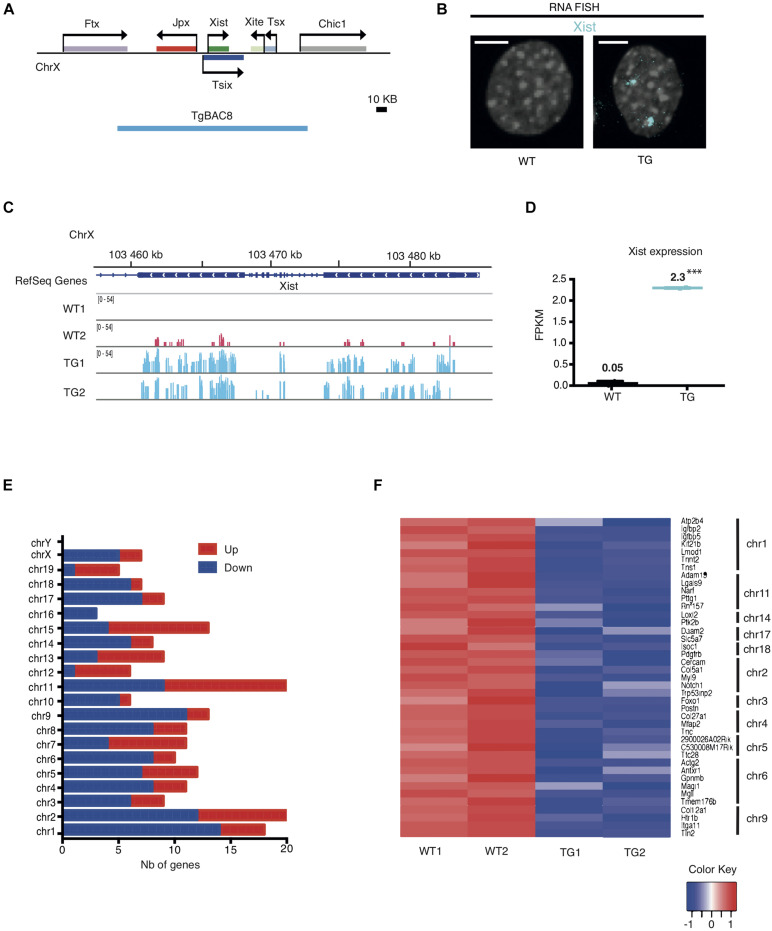
Integration of the *Xic* sequence in an autosome leads to differential gene expression in mouse fibroblasts. **(A)** Map of the *Xic* locus and the coverage of the 200 kb TgBAC8 (blue line), which includes the non-coding genes: *Xist*, *Jpx*, *Tsix, Xite*, and *Tsx*. The transgene construct TgBAC8 was used in generating transgenic mice as described in Sun et al. (2015). **(B)** *Xist* RNA FISH in male Tail Tip Fibroblasts (TTFs) isolated from wildtype and transgenic mice. The fluorescent Cy5-labeled Xist probe (cyan) detected *Xist* RNA as two “clouds” in the TG cell; no *Xist* RNA present in the WT cell. WT and TG male cells are tetraploid due to SV40 large T-transformation. The scale bar is 5 μm in length. **(C)** RNA-seq tracks showing *Xist* transcripts in WT and TG fibroblast samples in duplicate. **(D)** FPKM values for *Xist* transcripts in WT and TG fibroblast samples by RNA-seq. ^∗∗∗^*p* < 0.001. **(E)** Histogram depicting the number (Nb) of down- and up-regulated genes categorized by chromosomes in TG cells by RNA-seq. **(F)** Heatmap of individual genes with RNA-seq transcripts in WT and TG cell samples in duplicate. Down-regulated genes in TG samples that are less than 5 Mb apart within the same chromosome are shown. The color key box represents the log2 of the FPKM value.

Using transgenic mouse models, our previous work had defined a 200-kb *Xic* sequence including five lncRNAs (Figure 1A), which are sufficient to induce and maintain transgenic *Xist* expression from an autosome in both male and female mouse embryos (Sun et al., 2015). In this study, we analyzed the transcriptional consequence of the ectopic *Xist* expression in the transgenic mouse line Tg2087, which carries two copies of the *Xic* sequence integrated in a single autosomal locus. Hemizygous Tg2087/+ males are fertile, whereas homozygous Tg2087 males are sterile with defective sperm cells (Sun et al., 2015). We isolated tail-tip fibroblasts (TTFs) from the progeny of hemizygous Tg2087/+ crossed with wildtype, which produced equal frequencies of Tg2087/+ and +/+ offspring. A comparative gene expression analysis between transgenic and wildtype cells was performed using immortalized TTFs from Tg2087/+ and +/+ male littermates. The immortalized TTF cells were tetraploid due to SV40 Large T-transformation. As shown in Figure 1B visualizing *Xist* expression by RNA FISH, wildtype (WT) male cells showed no *Xist* signal, whereas the tetraploid Tg2087/ + male cells showed two *Xist* clouds. This confirms a stable transgenic *Xist* expression.

To investigate how ectopic *Xist* expression alters transcription of other genes, we performed total RNA sequencing on transgenic (TG) and wildtype (WT) samples in duplicate. Analysis of the RNA-seq results validated the *Xist* expression induced in transgenic male cells as compared to the wildtype controls. As shown in Figures 1C,D, WT male fibroblasts had no or only baseline expression of *Xist*. By contrast, TG male fibroblasts exhibited a significant increase of *Xist* transcripts. Comparative gene expression analysis identified differentially expressed coding and non-coding genes in TG as compared to WT. 130 genes were down-regulated and 83 genes were upregulated in TG (with adjusted *p* < 0.01 and Abs(logFC) ≥ 1). In particular, chromosomes 1, 2, and 9 were associated with relatively higher numbers of down-regulated genes with 14, 12, and 11 down-regulated genes, respectively (Figure 1E and Supplementary Table 1). Further analysis showed that Chr 1 carried the most number of down-regulated genes that are physically linked: the heatmap which displays any down-regulated gene that is less than 5 Mb apart from another down-regulated gene within the same chromosome showed seven genes were clustered in Chr 1, and five and four genes clustered in Chr 2 and Chr 9, respectively (Figure 1F). These results indicate that the autosomal *Xic* transgene in Tg2087/ + maintained active transcription of *Xist*, which affected genome-wide expression with specific silenced genes mostly clustered in Chr 1, Chr 2, and Chr 9.

Transcriptomic changes between TG and WT cells could be the result of both primary and secondary effects of transgene integration. To determine which chromosome was most affected by *Xist* transgene silencing, we applied three criteria: (1) the number of genes down-regulated within the chromosome, (2) the percentage of genes down-regulated out of all the genes within the chromosome, and (3) the number of down-regulated genes that are located in clusters within the chromosome. Using these three criteria, Chr 1 and Chr 9 were clearly the top candidates most affected by transgene silencing. Note that the transcriptional silencing on Chr 2 appeared much less significant once the total number of genes on the chromosome was taken into account. Specifically, 0.7% of the genes on Chr 2 were down-regulated as compared to 1.2 and 1.0% of genes down-regulated on Chr 1 and Chr 9, respectively (Supplementary Table 2). Additionally, we also considered a less stringent cutoff threshold of Abs(logFC) = 0.5) for RNA-seq analysis. This led to slightly higher numbers of genes shown as down-regulated across most of the chromosomes, and moreover, chromosomes 1, 8, 9 were ranked together at the top with the highest percentages of down-regulated genes (Supplementary Table 2). However, Chr 8 was excluded for further analysis since the down-regulated genes on Chr 8 had only three of them being clustered within the chromosome. In all, we concluded that chromosomes 1 and 9 were most affected by the *Xist* transgene silencing in the TG male fibroblasts.

### *Xic* Transgene Is Associated With Chromosome 1

**FIGURE 2:**
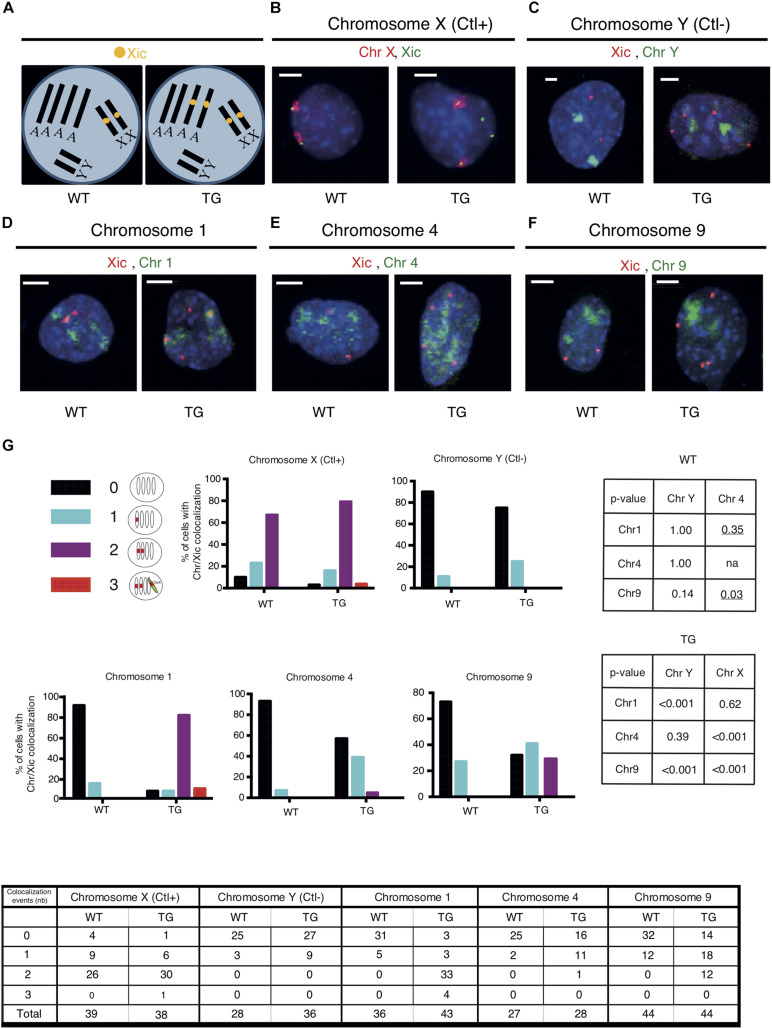
The *Xic* transgene co-localizes with chromosome 1 in TG male fibroblasts. **(A)** DNA FISH patterns are shown for Xic signals in WT and TG male TTFs with tetraploid chromosomes. There are four copies of each autosome and two copies of each sex chromosome. In WT cells, there are two copies of Xic signals corresponding to the endogenous *Xic*; in TG cells, a total of four Xic signals are present with two for the endogenous *Xic* and two for the *Xic* transgenes. **(B–F)** Dual-color DNA FISH was performed using chromosome paints in combination with Xic probes as indicated for WT and TG cells. Chromosome X was used as a positive control (Ctl+) for the endogenous *Xic* co-localizing with the X chromosome in both WT and TG cells; chromosome Y was used as a negative control (Ctl-) for the absence of co-localization between *Xic* and the Y chromosome in both WT and TG cells. Representative images are shown; the scale bar is 5 μm in length. **(G)** Summary of co-localization patterns and events. Numbers of co-localizations between Xic and chromosome paint signals within a cell nucleus are illustrated. Histograms depicting the percentage of cells with detected co-localization events are shown. *P*-values are shown in the tables for pairwise comparisons performed by Fisher’s Exact Test on the co-localization events in WT and TG samples: two-tailed *p*-values were used across all comparisons except where one-tailed *p*-values (underlined) were used when testing any increase of co-localization in the comparisons to Chr 4; na, not applicable. Cell numbers are listed in the chart.

Given the high occurrence of down-regulated genes clustered in Chr 1 and Chr 9 of Tg2087/+ cells, we examined whether the *Xic* transgene is integrated in either of the two chromosomes in Tg2087. To test the association of the *Xic* transgene with a specific chromosome, two-color DNA FISH was performed on Tg2087/+ cells using a combination of chromosome painting and *Xic* sequence-specific probes. Since the cells were tetraploids, probes for the *Xic* locus showed up as two spots in wildtype males and four spots in transgenic males (Figure 2A). Using chromosome X (Chr X) paint as a positive control, the dual-color DNA FISH showed the co-localization of two Chr X domains with two *Xic* sequence (Xic) spots in both WT and TG male cells: two additional Xic spots separated from Chr X were clearly present only in the TG cell (Figure 2B). By contrast, a negative control using chromosome Y (Chr Y) paint in combination with the Xic probes showed distinct localizations of two Chr Y domains in both WT and TG male cells: a total of four Xic spots were seen only present in the TG cell (Figure 2C). These control experiments validated the dual-color DNA FISH in not only detecting specific chromosomes but also determining their association with *Xic* in WT and TG cells.

To test whether the *Xic* transgene is associated with Chr 1 or Chr 9, we used chromosome paints specific for Chr 1 and Chr 9, which were each compatible with Xic probes for the dual-color DNA FISH on the Tg2087 TTF cells (Figures 2D,F). In addition, we used the chromosome paint specific for Chr 4 as a reference and control for any possible influence of chromosome size in resolving the DNA FISH signals combining Xic probes and chromosome painting (Figure 2E). Mouse chromosome 4 is 156.86 Mb, which represents the average size between mouse chromosome 1 (195.15 Mb) and mouse chromosome 9 (124.36 Mb) according to *Mus musculus* GRCm39 (Genome Reference Consortium Mouse Reference 39). The mouse chromosome paints (MetaSystems) specific for Chr 1, Chr 4, and Chr 9 were each compatible for hybridization together with the Xic probes in a dual-color DNA FISH process (Figures 2D–F). In WT cells, two Xic pinpoint signals corresponding to the two endogenous *Xic* loci on the two X chromosomes showed no obvious linkage to Chr 1, Chr 4, or Chr 9 chromosome paint signals. In TG cells, two out of the four copies of Xic pinpoint signals corresponded to the endogenous *Xic* and the other two Xic pinpoints corresponded to the transgenic *Xic*. As shown in Figure 2D, distinct associations between Xic pinpoints and chromosome paints were observed for two Xic signals and Chr 1 signals, consistent with the model illustrating *Xic* transgene in the autosome (Figure 2A). In comparison, associations between Xic pinpoints and the chromosome paints were much less obvious for Chr 4 and Chr 9 signals (Figures 2E,F), suggesting that the co-localization between Xic and chromosome paint signals is chromosome-specific and likely based on genetic linkage independently of the chromosome size.

An illustration of all co-localization patterns and quantitation of events observed are summarized in Figure 2G. The co-localization of three copies of Xic signals with chromosome paints observed in a few situations was likely due to transient proximity contacts between *Xic* and genetically unlinked chromosomal regions. Co-localization of two copies of Xic with chromosomes was observed in 77% of TG cells for Chr 1, very close to the co-localization observed for Xic with chromosome X in 79% of TG cells (*p* = 0.62). The distribution patterns observed thus suggest an association of Xic with Chr 1 very similar to the linkage of Xic with Chr X in the TG cells. As controls, in WT cells, the co-localization pattern of Xic with Chr 1 was the same as the pattern of Xic with chromosome Y (*p* = 1.000) and was significantly different from the pattern of Xic with chromosome X (*p* < 0.001). These data were consistent with the observation of endogenous *Xic* (on Chr X) separate from Chr 1 and Chr Y. Therefore, the strong co-localization of Xic with Chr 1 in TG cells indicates a linkage between the transgenic *Xic* and chromosome 1. By contrast, for Chr 4 and Chr 9, their co-localization with two copies of Xic appeared in only 4 and 27% of TG cells, respectively, both significantly less than the occurrence of co-localization between chromosome X and Xic (*p* < 0.001).

We noted that transient proximity contacts between a chromosome and the *Xic* sequence (endogenous or transgenic) would contribute to the observed colocalization events. In WT cells with the endogenous *Xic*, any co-localization between Xic and a non-X chromosome would indicate a transient proximity contact. As shown in Figure 2G, Xic co-localized with Chr Y in 11% of WT cells. In comparison, the frequency of such co-localization was very similar at 14% for Chr 1 (*p* = 1.00) and 7% for Chr 4 (*p* = 1.00) but appeared higher at 27% for Chr 9 (*p* = 0.14). Using Chr 4 as a reference for an autosome with its chromosome size ranked between Chr 1 and Chr 9, which both showed a higher frequency of co-localization with Xic in WT cells as compared to Chr 4, we noted that such co-localization events observed as expected for Chr 1 (one-tailed *p* = 0.35) but significantly more frequent for Chr 9 (one-tailed *p* = 0.03). These data suggest that transient proximity contacts occurred between the endogenous Xic and autosomes; while Chr 1 and Chr 4 exhibited similar frequencies at close distances with the endogenous *Xic*, Chr 9 had more frequent proximity contacts with *Xic*.

In TG cells with the Xic probes detecting both endogenous and the transgenic *Xic*, the co-localization between Xic and chromosome paint signals reflects both the genetic linkages and the transient proximity contacts. The co-localization frequency of Xic/Chr 1 in TG cells was comparable to that of Xic/Chr X, both of which were significantly higher than that of Xic/Chr 4 or Xic/Chr 9. Our overall observations thus support a genetic linkage between *Xic* and chromosome 1 equivalent to *Xic* and chromosome X. A high frequency of Xic/Chr 9 co-localizations observed in TG cells, as compared to that of Xic/Chr 4, was likely contributed by the frequent proximity contact between chromosome 9 and the *Xic* locus.

### *Xic* Transgene Represses Expression of Genes on Chr 1 With Close Spatial Proximity

**FIGURE 3:**
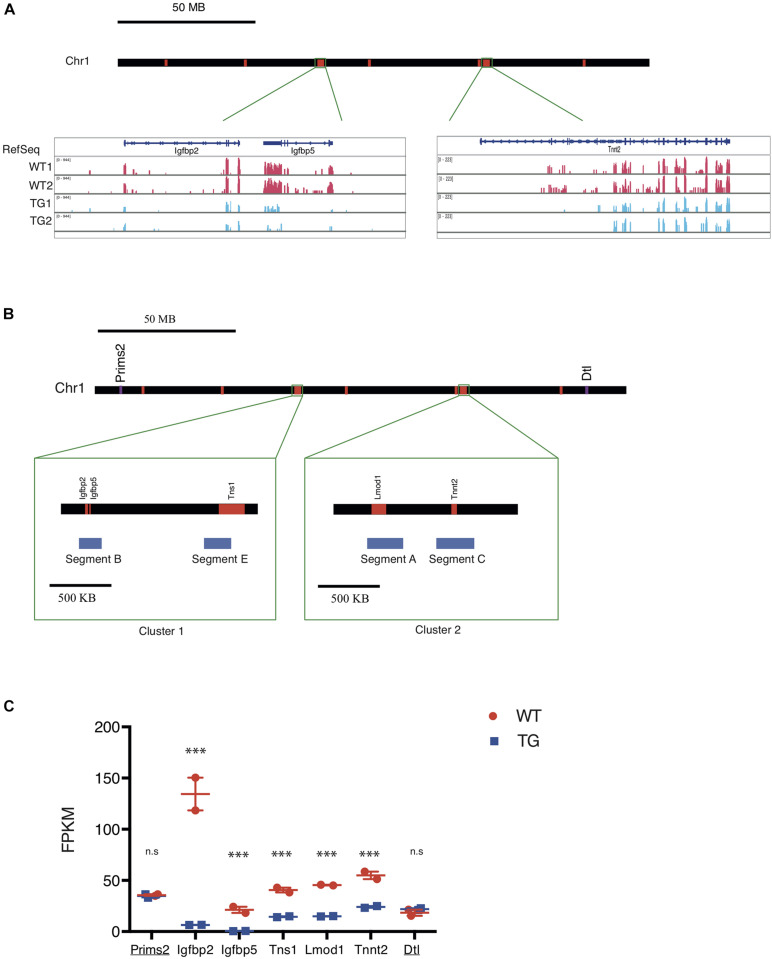
The *Xic* transgene induces transcriptional repression of genes clustered on chromosome 1. **(A)** Map of mouse chromosome 1 with regions of down-regulated genes labeled in red. Representative RNA-seq tracks showing transcripts in WT and TG fibroblast samples for three genes, *Igfbp2, Igfbp5*, and *Tnnt2*, which are located in two chromosomal regions. **(B)** Map of mouse chromosome 1 showing two clusters of down-regulated genes. Cluster 1 spans Chr1: 72, 824, 480-74, 124, 449; Cluster 2 spans Chr1: 135, 301, 535-136, 178, 014. Within Cluster 1, Segment B and Segment E correspond to transcriptionally repressed genes *Igfbp2*/*Igfbp5* and *Tns1*, respectively; whereas in Cluster 2, Segment A and Segment C correspond to transcriptionally repressed genes *Tnnt2* and *Lmod 1*, respectively. Two genes, *Prims2* and *Dtl* (labeled in purple), are located distal to the down-regulated genes (labeled in red) arranged in the chromosome. **(C)** FPKM values for the seven genes along the chromosome with their expression detected in WT (red) and TG (blue) cell samples by RNA-seq. *Prims2* and *Dtl* (underlined) are located outside of Clusters 1 & 2 that include *Igfbp2*, *Igfbp5*, *Tns1*, *Lmod1*, and *Tnnt2*. n.s., not significant; ^∗∗∗^*p* < 0.001.

Gene expression analysis from RNA-seq showed two major clusters of down-regulated genes from Chr 1 in TG cells when compared with WT controls (Figure 3A). This suggested to us that the *Xic* transgene may be integrated proximally to one of these two clusters in Chr 1. To test this, we designed DNA FISH probes targeting the sequence segments (170–210 kb) within the two transcriptionally repressed clusters in Chr 1 (Figure 3B): Segments B and E are 800 kb apart within the 1.5 Mb Cluster 1; Segments A and C are 250 kb apart within the 1.1 Mb Cluster 2. The chromosomal distance between Cluster 1 and Cluster 2 is 60 Mb. Among the transcriptionally repressed genes in TG cells, the Insulin-like growth factor binding protein genes *Igfbp2* and *Igfbp5* are located in Segment B; the actin-binding Tensin protein gene *Tns1* is in Segment E; the actin filament pointed end-capping protein Leiomodin 1 gene *Lmod1* is in Segment A; the cardiac muscle troponin T (cTnT) protein gene *Tnnt2* is in Segment C (Figure 3B). Among these five genes, *Igfbp2* and *Igfbp5* were most affected by the transgene, with an expression reduction of >95% in TG cells compared to WT controls; *Lmod1* expression was reduced by 69%, with *Tns1* expression reduced by 66% and *Tnnt2* expression reduced by 58%, in TG cells (Figure 3C and Supplementary Table 1). By contrast, the DNA primase large subunit gene *Prims2* is located 60 Mb distal of Cluster 1 with no expression change in the TG cells compared to WT controls. Similarly, the denticleless E3 ubiquitin protein ligase homolog gene *Dtl* is located 60 Mb distal of Cluster 2 and its expression was not affected by the transgene (Figure 3C).

**FIGURE 4:**
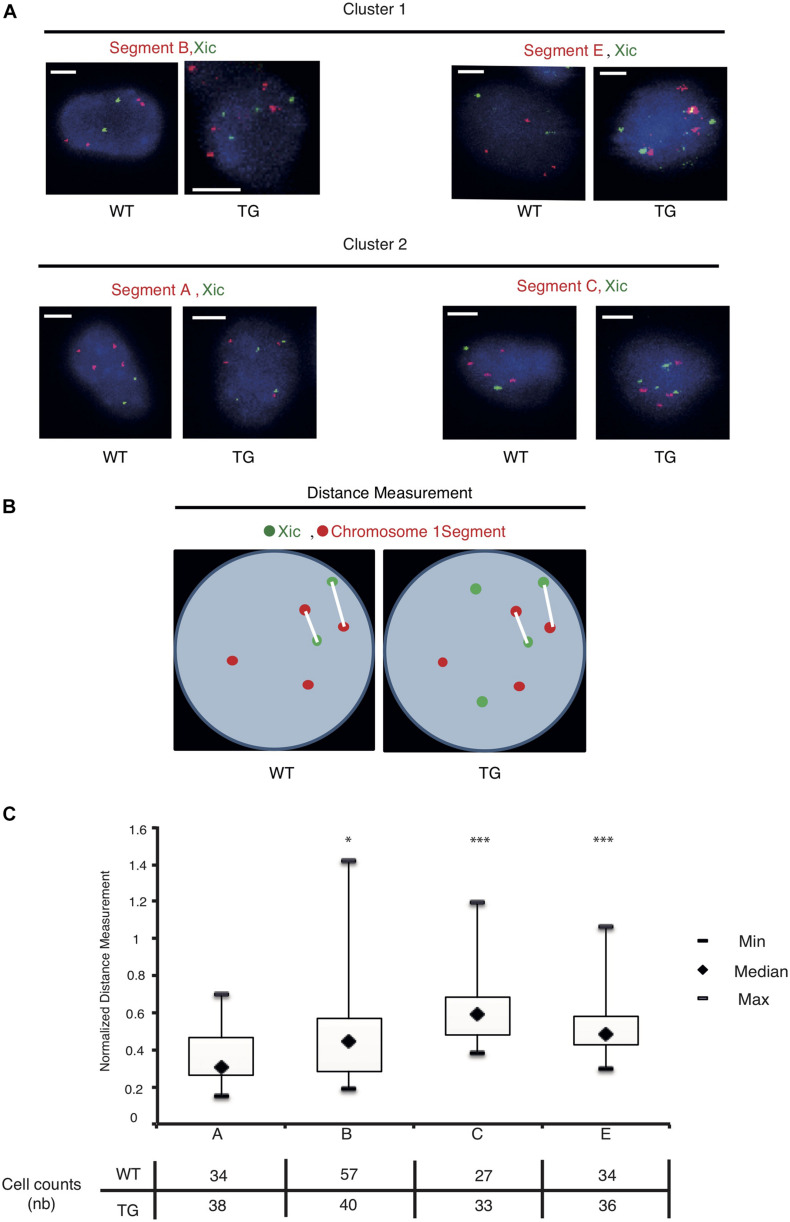
The *Xic* transgene is in spatial proximity with the transcriptionally repressed sequence segments on chromosome 1. **(A)** Dual-color DNA FISH is performed using chromosome 1 segment probes in combination with Xic probes as indicated for WT and TG TTFs. Representative images are shown; the scale bar is 5 μm in length. **(B)** Measurements of distance between the chromosome segment and Xic signals are illustrated with two shortest distances at display for each cell nucleus. **(C)** Boxplots of the normalized measurements for spatial distances between *Xic* and segments A, B, C, E, in TG cells. The table under the box plot shows the number of cells analyzed for each segment. ^∗^*p* < 0.05, ^∗∗∗^*p* < 0.001, by independent 2-group *t*-test as compared with Segment A.

We used the dual-color DNA-FISH to map the nuclear positions of the sequence segments relative to *Xic* inside the cell (Figure 4A). In the WT cell, two copies of Xic signals corresponded to the endogenous *Xic* sequence from two X chromosomes in the tetraploid male cell, together with four copies of the “Segment” signals from four Chr 1’s. In the TG cell, four copies of Xic signals were present, two from the endogenous genes and two from the transgenes, with four copies of the “Segment” sequence signals. As illustrated in Figure 4B, we measured the spatial distance between an Xic signal spot and a “Segment” signal spot within the three-dimensional nucleus, and recorded the two shortest straight-line distances among all pairwise measurements for each cell. In the WT control cell, the shortest distances between “Segment” and Xic represent the spatial proximity of the Chr 1 segment sequences to the endogenous *Xic*, when they become or are closely localized in the cell nucleus. In the TG cell, the shortest distances between “Segment” and Xic represent the spatial proximity of the Chr 1 segment to both the endogenous and the transgenic *Xic*. To compare the nuclear positions of four Chr 1 segments relative to *Xic* in the TG cell, we normalized the distance measurements in TG cells against the same measurements in the WT control cell. In doing so, we eliminated the bias due to any close transient associations between Chr 1 segment sequences and the endogenous *Xic*.

**FIGURE 5:**
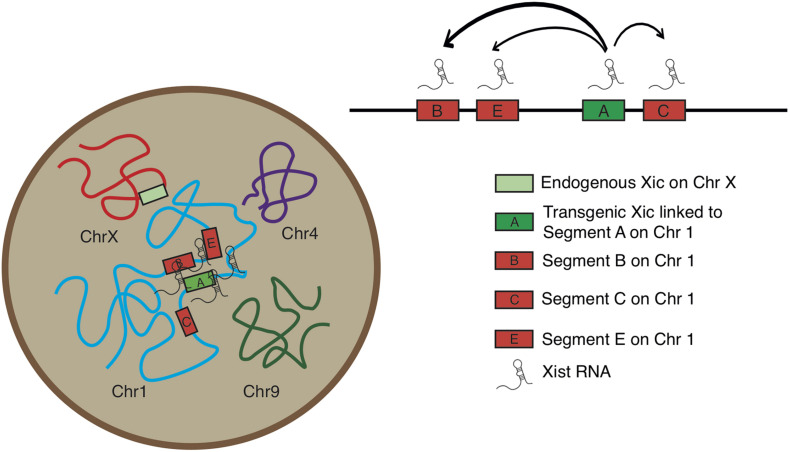
Model for *Xic* transgene activating *Xist* RNA and silencing chromosome 1 segments facilitated by three-dimensional chromosomal configuration.

Analysis of the normalized measurements showed that *Xic* had the closest spatial proximity to Chr 1 Segment A in the TG cell (Figure 4C). The comparisons of distance distributions illustrated that the spatial proximity between Segment A and *Xic* was significantly closer than the spatial distance between Segment C and *Xic*, even though Segment A and Segment C are both within the same Cluster 1. In contrast, Segment E is located in Cluster 2 and separated from Segment C with a large chromosomal distance of >60 Mb in between, however, displayed comparable spatial distance to *Xic* in the TG cell as Segment C. We noted that Segment B located in Cluster 2 showed the most variable spatial distance from *Xic*, with a median value close to the value of Segment A from *Xic*, even though Segment B is >60 Mb chromosomal distance away from Segment A. These results illustrate a model that *Xist* expressed from the *Xic* transgene on Chr 1 is capable of accessing various locations on Chr 1 for silencing, and that *Xist* RNA induces autosomal gene silencing by searching in three dimensions for sequences of close proximity (Figure 5).

## Discussion

*Xist* RNA and XCI are considered paradigms for studying epigenetic regulation and mechanisms of chromatin organization (Jégu et al., 2017; Galupa and Heard, 2018; Fang et al., 2019). Experimental models incorporating transgenes of the *Xic* sequence are foundational in defining the functional elements and mechanisms of XCI as previous studies have shown that the integration of *Xic* in an autosomal region can lead to *Xist* RNA expression (Heard et al., 1996; Lee and Jaenisch, 1997; Sun et al., 2015; Loda et al., 2017). Our results here using tail tip fibroblasts derived from *Xic* transgenic mice provide evidence for stable *Xist* expression induced by the *Xic* sequence integrated in chromosome 1. We have shown that the transgenic *Xist* expression causes transcriptional reduction of genes clustered in chromosome 1, suggesting chromosome silencing effect of the transgene. Moreover, by correlating the chromosome positions of sequence regions with their spatial distances in the cell nucleus, we have demonstrated that the silencing effect of transgenic *Xist* can extend to chromosome 1 regions across a physical chromosome distance of 60 Mb, and that three-dimensional chromosome conformation facilitates the proximity contacts of *Xist* RNA to access sequences across a large chromosome distance.

Three-dimensional organization of the chromosome and close proximity contacts have been identified as essential factors affecting *Xist* RNA spreading and chromosome silencing during XCI (Engreitz et al., 2013; Simon et al., 2013; Wang et al., 2019). In these studies, functional assays including RNA Antisense Purification (RAP), Capture Hybridization Analysis of RNA Targets (CHART), and Chromosome Conformation Capture (Hi-C) were used in combination with parallel sequencing for high-resolution maps of RNA-chromatin and chromatin-chromatin spatial interactions in established mouse cell lines. This current study took advantage of our previously established *Xist* transgenic mice and investigated the *Xist*-induced chromosome silencing in male mouse fibroblasts. The performed dual-color DNA FISH and image analysis allowed us to detect the spatial interactions between *Xist* and specific chromosomal regions at the single-cell level. The results obtained are consistent with the understanding that chromosome silencing by *Xist* is not a one-dimensional linear effect but is associated with three-dimensional spatial chromatin configuration and distances.

The transgenic mouse lines that we had previously generated incorporated the *Xic* sequence into autosomes to test whether a change in the flanking sequence of *Xist* would affect chromosome silencing. In fact, all autosomal *Xist* transgenes continued to express *Xist* and induce silencing of that autosome in mice (Sun et al., 2015; Carmona et al., 2018). However, it remained unclear how the non-coding RNA genes within the *Xic* locus (Figure 1A) interact and work together in the XCI process for mouse chromosomes. The transgenic mouse model has enabled us to analyze the *Xic* activity *in vivo* without affecting the endogenous *Xic* function in X-chromosome silencing and dosage compensation, which is essential for animal survival. As shown in the RNA-seq results here for Tg2087/+, five genes on chromosome X exhibited down-regulation in the TG male cell (Figure 1E). However, these genes were not clustered. Among the X-linked genes, the Sushi repeat-containing protein gene *Srpx2* was transcriptionally reduced by >95% (Supplementary Table 1). Although this gene does not affect mouse viability, the functional roles of *Srpx2* have been implicated in glutamatergic synapse formation and its mutations affect ultrasonic vocalizations in mouse pups (Sia et al., 2013). Therefore, transgenic *Xist* expression in autosomes could affect individual X-linked gene activities and lead to secondary phenotypic consequences.

Compared to chromosome 21 in human cells, which could be efficiently silenced by an inducible *XIST* transgene (Jiang et al., 2013), chromosome 1 in our transgenic mouse cells with a stably inserted *Xic* sequence did not show whole-chromosome silencing. Mouse chromosome 1 has around 2000 genes, and only 14 were significantly down-regulated, suggesting that the efficiency of *Xist*-induced chromosomal silencing can vary in different autosomes. Our results are, however, consistent with previously reported *Xist* transgenes on long-range silencing in autosomes, where epigenetic and genetic features like CTCF density, LINEs and SINEs may affect the silencing efficiency (Tang et al., 2010; Loda et al., 2017). As shown in Supplementary Figure 1, mouse X chromosome has a higher density of LINEs than mouse chromosome 1 while the SINEs density appears comparable between the two chromosomes, consistent with the previous findings on LINEs/SINEs patterns and effects (Loda et al., 2017). Similarly in humans, the X chromosome also has a higher density of LINEs than chromosome 21, suggesting that additional factors such as small chromosome size may have contributed to the high-efficiency of *XIST*-mediated silencing in the Down syndrome model. The *XIST* transgene insertion site also likely played a role in the observed autosomal silencing efficiency, as autosomal flanking regions can impact the recruitment of heterochromatin marks. And lastly, it has also been noted that *XIST*-induced chromosomal silencing becomes less efficient after cell differentiation (Kelsey et al., 2015).

We also point out here that three other situations may have contributed to the low silencing efficiency of the *Xist* transgene integrated in chromosome 1 of Tg2087/+ male fibroblasts: (1) the transgenic *Xist* expression is driven by its native promoter and the expression level in the male fibroblasts was lower than any inducible transgene expression or the endogenous *Xist* in female cells; (2) the TTFs are fully differentiated mouse cells that were isolated from the transgenic animals, which may have developed transcriptional compensation in the presence of the transgene; (3) other chromosomes (e.g., Chr 9) may be competing with chromosome 1 in the spatial proximity space for close contacts with *Xist* RNA. In order to investigate these effects, high-resolution mapping of the transgene integration sequence and functional studies of RNA-chromatin and chromatin-chromatin interactions would need to be performed. The high-throughput sequencing of transcriptomes with allelic differences will enhance the resolution and accuracy of the outcomes. In particular, the Tg2087 mouse line is maintained in a C57BL/6J strain background. To introduce known SNPs for allelic distinctions, F1 hybrids would be obtained from crosses between Tg2087-C57BL/6J (of *M. m. musculus*) and CAST/EiJ (of *M. m. castaneus*). Afterward, CHART-seq would be performed in combination with *in situ* Hi-C to map the *Xist* interacting chromatins and the spatial chromatin-chromatin interactions in the TG cells.

In general, our data supports a model that *Xist* silencing is dependent on the three-dimensional chromosomal architecture. It has also been previously noted that XCI involves multiple parallel pathways and cooperation among various factors. For example, YY1 and HnrnpU facilitate *Xist* RNA localization, and the polycomb repressive complexes (PRC1 and PRC2) are the central epigenetic regulators recruited to the inactive X chromosome (Lu et al., 2017; Colognori et al., 2019; Monfort and Wutz, 2020). Understanding how these factors are used by *Xist* in silencing autosomal sequences and also the direct comparison of endogenous and transgenic *Xic* will no doubt further deepen our knowledge of RNA-mediated regulation in epigenetic changes.

## Data Availability Statement

The datasets presented in this study can be found in online repositories. The sequencing data have been deposited in NCBI’s Gene Expression Omnibus (GEO) with the Accession number GSE 156393. Additional data information can be found in the article/Supplementary Material.

## Author Contributions

BL, C-HW, and SS designed the research. C-HW and SJ performed the RNA-seq experiment under the supervision of AM. IN, SJ, and BL performed the RNA-seq data analysis. BL performed the FISH experiments. IN and BL performed the image and data analyses. SC provided the fibroblast cell lines from transgenic mice, reagents for FISH, and illustration for Figure 5. AM provided the reagents and analytic tools for the RNA-seq analyses. C-HW and WL contributed in drafting the manuscript. IN, BL, and SS wrote the manuscript with input from all authors.

## Conflict of Interest

The authors declare that the research was conducted in the absence of any commercial or financial relationships that could be construed as a potential conflict of interest.

## Footnotes

1 https://bacpacresources.org/

## Supplementary Material

Supplementary Figure 1 LINEs and SINEs in mouse and human chromosomes. LINEs density **(left graph)** and SINEs density **(right graph)** are plotted as the percentages of the sequence occupied in mouse chromosomes X (ChrX) and 1 (Chr 1), and in human chromosomes X (ChrX) and 21 (Chr21). Data were downloaded from UCSC genome browser with Repeat Masker for mm10 genome and hg38 genome.
