# Supplementary figures and images for: Linking Chromosomal Silencing With Xist Expression From Autosomal Integrated Transgenes
Source: Front Cell Dev Biol. 2021 Jun 18;9:693154. doi: 10.3389/fcell.2021.693154 (PMC8250153; doi:10.3389/fcell.2021.693154)

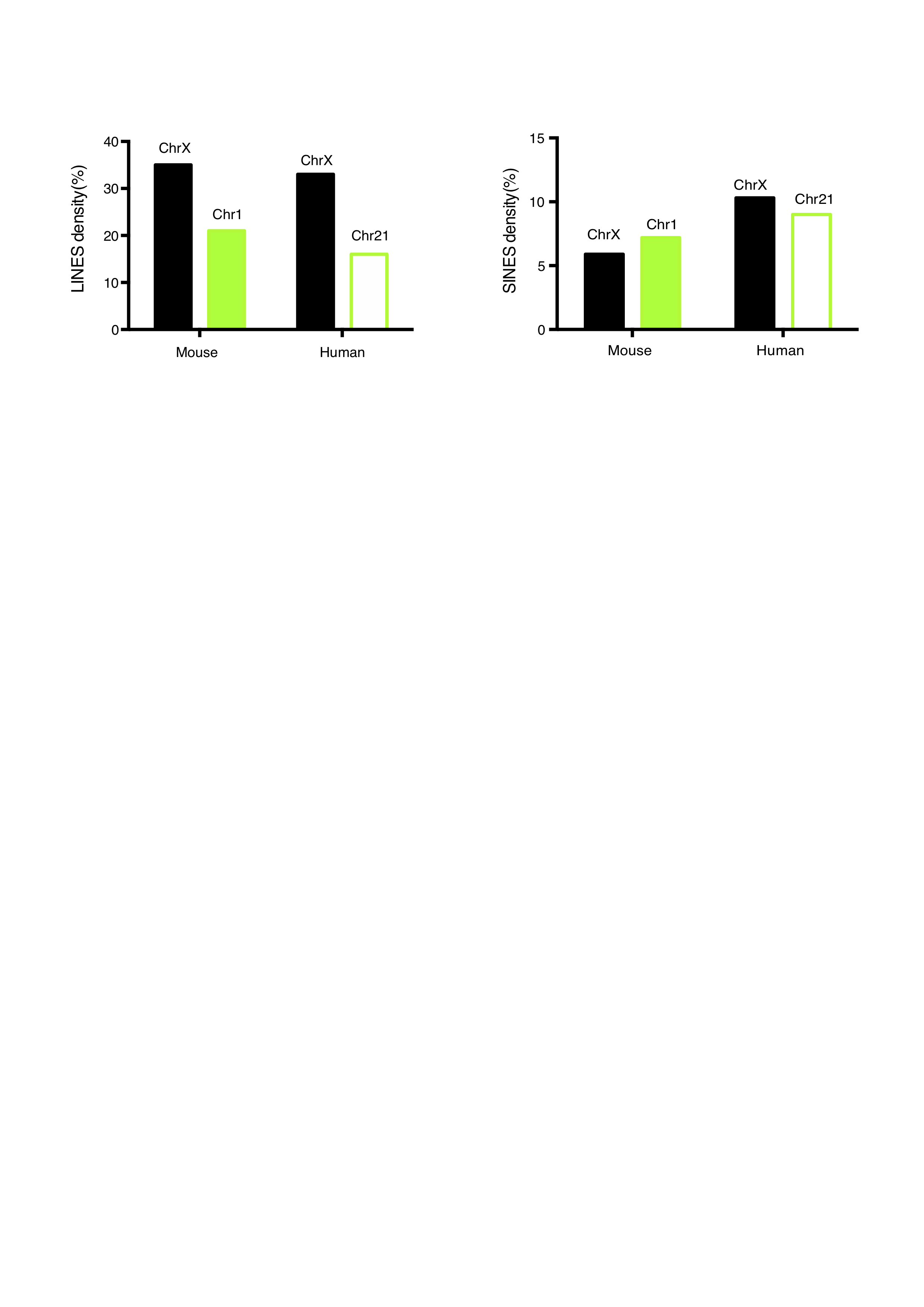

Supplement: Supplementary Figure 1 — LINEs and SINEs in mouse and human chromosomes. LINEs density (left graph) and SINEs density (right graph) are plotted as the percentages of the sequence occupied in mouse chromosomes X (ChrX) and 1 (Chr 1), and in human chromosomes X (ChrX) and 21 (Chr21). Data were downloaded from UCSC genome browser with Repeat Masker for mm10 genome and hg38 genome. [file Image_1.JPEG]
